# Epigenetic Modifications and Non-Coding RNA in Diabetes-Mellitus-Induced Coronary Artery Disease: Pathophysiological Link and New Therapeutic Frontiers

**DOI:** 10.3390/ijms23094589

**Published:** 2022-04-21

**Authors:** Francesca Romana Prandi, Dalgisio Lecis, Federica Illuminato, Marialucia Milite, Roberto Celotto, Stamatios Lerakis, Francesco Romeo, Francesco Barillà

**Affiliations:** 1 Division of Cardiology, Department of Systems Medicine, Tor Vergata University, 00133 Rome, Italy; dalgisio.lecis@gmail.com (D.L.); feilluminato@gmail.com (F.I.); mari.milite@gmail.com (M.M.); celott.roberto@yahoo.it (R.C.); francesco.barilla@uniroma2.it (F.B.); 2 Department of Cardiology, Mount Sinai Hospital, Icahn School of Medicine at Mount Sinai, New York, NY 10029, USA; stamatios.lerakis@mountsinai.org; 3 Department of Departmental Faculty of Medicine, Unicamillus-Saint Camillus International University of Health and Medical Sciences, 00131 Rome, Italy; romeocerabino@gmail.com

**Keywords:** diabetes mellitus, hyperglycemia, coronary artery disease, epigenetics, epidrugs

## Abstract

Diabetes mellitus (DM) is a glucose metabolism disorder characterized by chronic hyperglycemia resulting from a deficit of insulin production and/or action. DM affects more than 1 in 10 adults, and it is associated with an increased risk of cardiovascular morbidity and mortality. Cardiovascular disease (CVD) accounts for two thirds of the overall deaths in diabetic patients, with coronary artery disease (CAD) and ischemic cardiomyopathy as the main contributors. Hyperglycemic damage on vascular endothelial cells leading to endothelial dysfunction represents the main initiating factor in the pathogenesis of diabetic vascular complications; however, the underlying pathophysiological mechanisms are still not entirely understood. This review addresses the current knowledge on the pathophysiological links between DM and CAD with a focus on the role of epigenetic modifications, including DNA methylation, histone modifications and noncoding RNA control. Increased knowledge of epigenetic mechanisms has contributed to the development of new pharmacological treatments (“epidrugs”) with epigenetic targets, although these approaches present several challenges. Specific epigenetic biomarkers may also be used to predict or detect the development and progression of diabetes complications. Further studies on diabetes and CAD epigenetics are needed in order to identify possible new therapeutic targets and advance personalized medicine with the prediction of individual drug responses and minimization of adverse effects.

## 1. Introduction

Diabetes mellitus (DM) is a glucose metabolism disorder characterized by chronic hyperglycemia resulting from a deficit of insulin production and/or action. DM affects 537 million adults between the age of 20–79 years worldwide (10.5% prevalence), and this number is predicted to rise to 783 million by 2045 (12.2% prevalence) [1]. Over 1.2 million children and adolescences have type 1 DM (T1DM). It is estimated that 240 million people are living with undiagnosed diabetes, mainly in low- and middle-income countries [2].

T1DM accounts for about 5–10% of all patients with DM, and it results from pancreatic beta cells dysfunction with reduced insulin secretion, while type 2 DM (T2DM) is related to insulin resistance and accounts for 90–95% of all diabetic patients [3]. Diabetes is a major driver of mortality worldwide. Approximately 6.7 million adults are estimated to have died globally from diabetes or its complications in 2021 (12.2% of all deaths) [2]. 

DM significantly increases the risk of cardiovascular diseases (CVD), all-cause mortality and cardiovascular mortality. According to the Framingham study, patients with DM have a two-fold to four-fold increased risk of developing coronary artery disease (CAD) and myocardial infarction (MI) and a four-fold to six-fold increased risk of developing congestive heart failure (HF) [4]. Hyperglycemia represents the main initiating factor in the pathogenesis of diabetic complications [5]. 

HbA1c is the index of mean glycemia over time, and it is widely used for the routine monitoring of long-term glycemic control in both T1DM and T2DM. The normal reference range for HbA1c is 20–40 mmol/mol (4–6%); however, the general target for the treatment of diabetic patients is <53 mmol/mol (<7%) [6]. Patients with DM and poor glycemic control have a cardiovascular mortality risk that is ten-fold higher in subjects with T1DM [7] and five-fold higher in patients with type 2 DM (T2DM) [8]. CVD accounts for two thirds of overall deaths in subjects with T2DM, with CAD and ischemic cardiomyopathy as the main contributors.

People living with DM are at risk of developing several debilitating complications, leading to an increased need for medical therapy and hospitalizations, reduced quality of life and increased health expenditures. Global diabetes-related health expenditures were estimated at 966 billion USD in 2021 and are predicted to reach 1054 billion USD by 2045 [1].

Diabetic heart disease includes CAD, cardiac autonomic neuropathy (CAN) and diabetic cardiomyopathy. CAD is a well-known cardiac complication of DM, while CAN and diabetic cardiomyopathy are often under-recognized and undiagnosed. CAN is characterized by structural and functional alterations in the myocardial innervation that result in damage to the cardiac autonomic nerves, leading to orthostatic hypotension, reduced exercise tolerance and non-dipping/reverse-dipping at night [9].

Diabetic cardiomyopathy is a DM-induced pathophysiological condition characterized by cardiac structural, functional and metabolic changes that can lead to HF in the absence of CAD, hypertension and valvular heart disease. Structural changes in diabetic cardiomyopathy are mainly represented by cardiac stiffness, hypertrophy and fibrosis that eventually lead to HF with preserved ejection fraction (EF) (HFpEF, restrictive phenotype) and/or HF with reduced EF (HFrEF, dilated phenotype) [10].

The phenotypes and underlying mechanisms of diabetic heart disease have been mostly investigated in animals and humans with T2DM. The impact of T1DM is less clear, because the results of human studies are controversial and the metabolic derangements and the phenotype may be attenuated or masked by insulin treatment, since glycemic control reduces the prevalence of diabetic cardiomyopathy and of CVD [10]. In T1DM, diabetic cardiomyopathy is characterized by preserved systolic function, the absence of cardiac hypertrophy [11] and increased cardiomyocyte autophagy compared to T2DM [12]. These phenotypic differences may be explained by differences in myocardial insulin action, since T1DM is characterized by insulin deficiency while T2DM by insulin resistance (IR) with hyperinsulinemia, and this could have effects on cell survival, cell growth and other cellular pathways [10].

## 2. Diabetes Mellitus and CAD

DM is a strong pro-atherogenic stimulus, as suggested by the frequent involvement of multiple vascular districts. The risk of advanced atherosclerosis is five-fold higher in T2DM patients than in nondiabetic subjects [13] with a several-fold increased risk of coronary heart disease, cerebrovascular disease and peripheral vascular disease [4]. Diabetes is associated with a marked increase (by a factor of two to four) [4] in the risk of coronary heart disease, and it is considered as a CAD risk equivalent. This means that diabetic patients without a previous myocardial infarction have a risk of myocardial infarction as high as nondiabetic patients with previous myocardial infarction [14]. In diabetic patients, CAD is usually characterized by multivessel involvement with rapid disease progression and lesions that often require revascularization [9]. 

Patients with CAD and diabetes undergoing revascularization with drug-eluting stents present double rates of MI, stent thrombosis and cardiovascular mortality compared to non-diabetic patients [15]; moreover, they present 86% higher rates of in-stent restenosis. Restenosis and DM are independently associated with higher likelihood of death after 4-years [16]. Coronary artery bypass grafting (CABG) has been considered to be the preferred revascularization strategy in patients with DM and multivessel or complex CAD. However, in patients with left main coronary artery (LMCA) disease, the 10-year rates of mortality and serious composite outcomes were similar between percutaneous coronary intervention and CABG in patients with or without DM, suggesting that DM should not penalize the specific LMCA revascularization strategy [17].

In diabetic patients, CAD lesions have more complex and vulnerable features (lipid-rich core, macrophage accumulation and thin fibrous-cap) [9]. At the time of their first acute coronary syndrome, T2DM patients showed more severe and extensive coronary atherosclerosis, and this may be due to enhanced collateral circulation and more calcific plaques with less lipid deposition in the culprit vessel [18].

## 3. Pathophysiology of CAD in Diabetes Mellitus

Diabetes is a complex metabolic process that induces atherosclerosis development and/or accelerates its progression through a multifactorial process. Vascular complications associated with hyperglycemia in diabetes often begin with endothelial dysfunction; however, the mechanisms underlying the pathogenesis of CAD in diabetic patients are still not completely understood [19]. Most of the reversible risk factors for atherosclerosis are more prevalent among diabetic patients, including hypertension, obesity, dyslipidemia, hyperglycemia and hyperinsulinemia [20]. 

However, the contribution of the higher prevalence of all these classic risk factors in terms explains only a portion (25%) of the excess of coronary heart disease risk in T2DM [21]. This discrepancy is in part related to changes in the traditionally measured risk factors that occur in diabetes, including glycation and advanced glycation end products (AGEs), glycoxidation and oxidation, insulin resistance and hyperinsulinemia, abnormalities of apoprotein and lipoprotein particle distribution and procoagulant states [20]. Other factors to be considered are the possible adverse effects of hypoglycemic agents, the influence of diabetic cardiomyopathy, inflammation, genetic and epigenetic modifications.

Inflammation plays an important role in the pathophysiological link between DM and atherosclerosis. Vircow first recognized the role of inflammation in the pathogenesis of atherosclerosis [22]. Chronic inflammation and inflammasome activation play important roles in the pathogenesis of T2DM. Patients with T2DM have significantly increased messenger RNA (mRNA) and protein levels of the NLRP3 inflammasome and increased proinflammatory cytokines in monocyte-derived macrophages [23]. Patients with DM present more intense pro-inflammatory, pro-oxidant and pro-thrombotic stimuli; the latter is related to hyper-reactive platelets, the upregulation of pro-thrombotic markers and suppression of fibrinolysis [24].

Jarrett in 1984 [25] and Stern, in 1995 [26], suggested that DM and atherosclerosis have a common genetic and environmental background, rather than one being a complication of the other (the “common soil” hypothesis). Insulin-resistance and hyperglycemia, inflammation, oxidative stress, hypercoagulability, high blood pressure, dyslipidemia and obesity are common pathophysiological factors in T2DM and CVD. Many common single-nucleotide polymorphisms (SNP) have been associated with an increased risk of CVD and T2DM [27]. 

Genome-wide association studies have been employed to search for gene predispositions to both diseases. The main genes whose variants are commonly associated with both T2DM and CVD are involved in LDL oxidation (paraxonase, polymorphism Gln-Arg 192 [28] or Met-Leu 54 [29], which lead to decreased levels or activity of paraxonase, an enzyme that normally protects LDL from proatherogenic modifications), redox balance (superoxide dismutase 2 and polymorphism Ala-Val 16, which lead to decreased SOD2 activity) [30], the adiponectin pathway (adiponectin, polymorphism +276 G/T [31] and adiponectin receptor ADIPOR1, haplotypes rs7539542, rs10920531 and rs4950894 [32]. 

These polymorphisms determine the reduced activity of the adiponectin pathway that normally has anti-inflammatory and antiatherogenic effects), lipoprotein transport (ApoE, polymorphism Arg112/Arg158 that encodes apoE4 isoform, which has a higher affinity for LDL-R, leading to early receptor occupation, accumulation of LDL particles, LDL-R synthesis suppression and lower clearance of lipoproteins from the body through LDL-R) [33] but also cell cycle regulation (CDKN2A/2B, variant rs4977574) [34], cell growth, differentiation and glucose metabolism (HMGA1, variant rs146052672, which is associated with increased susceptibility to T2DM and acute MI) [35]. 

A proteomic analysis of a T2DM population in relation to coronary phenotyping highlighted the potential role of GDF15, renin, adiponectin, serine protease HTRA1 and tetranectin in the development of T2DM and CAD [36]. There are also some genes with polymorphisms that are associated with a reduction of risk for CVD and T2DM, such as genes involved in the cholesterol metabolism (PCSK9), cells mitosis (PSRC1) or intracellular trafficking (SORT1) [27]. In addition to biochemical mechanisms and genetic factors, epigenetic mechanisms play a key role in the physiopathology of DM-induced CAD.

### 3.1. Role of Lipid and Glucose Metabolism in the Pathophysiological Link between Diabetes and Atherosclerosis

DM is a glucose metabolism disorder characterized by chronic hyperglycemia resulting from a deficit of insulin production and/or action. Both T1DM and T2DM cause hyperglycemia, which leads to endothelial dysfunction through different pathways. In addition, T2DM also causes insulin resistance, which is another determinant for endothelial dysfunction. Obesity, which is an independent risk factor for endothelial cell dysfunction is also closely related to type 2 diabetes [37].

**Figure 1:**
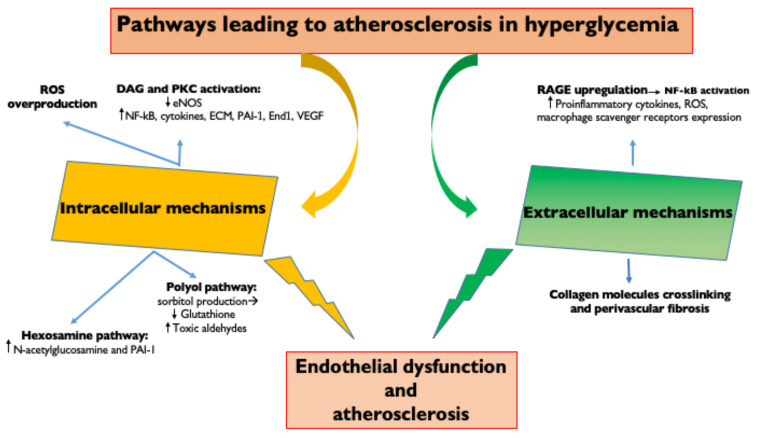
Pathways leading to atherosclerosis in hyperglycemia. Intracellular and extracellular molecular mechanisms underlying hyperglycemia-induced endothelial dysfunction. ROS, Reactive oxygen species; DAG, diacylglycerol; PKC, Protein kinase C; eNOS, endothelial Nitric Oxide Synthase; NFkB, Nuclear Factor κB; ECM, extracellular matrix; PAI—1, Plasminogen activator inhibitor-1; End1, endothelin 1; VEGF, Vascular endothelial growth factor; AGE, Advanced glycation end products; and RAGE, receptor for advanced glycation end products.

Hyperglycemia represents the main initiating factor in the pathogenesis of diabetic complications [5] (Figure 1). A causal relationship between levels of hyperglycemia and diabetic microvascular and macrovascular complications was found in both T1DM and T2DM patients [38]. Intensive diabetes therapy and lower levels of HbA1c in T1DM are associated with thinner carotid IMT, less coronary calcification and a lower incidence of clinical cardiovascular events, such as myocardial infarction, stroke and cardiac death [39]. In T2DM patients, metabolic control (HbA1c < 7%) reduces the risk of coronary heart disease death and all coronary heart disease events [40]. Glycemic control is therefore of fundamental importance to reduce the progression of diabetes and its microvascular and macrovascular complications.

Vascular endothelial cells are a major target of hyperglycemic damage; however, the mechanisms underlying this damage are still incompletely understood. Hyperglycemia is linked to atherosclerosis through both extracellular and intracellular mechanisms.

The extracellular mechanisms are based on the fact that chronic hyperglycemia induces the non-enzymatic glycation of proteins, lipids and lipoproteins with the generation of AGEs [41]. AGEs upregulate their receptors (RAGE) leading to the activation of transcription factors, such as nuclear factor-κB (NF-κB) and its target genes [41]. This results in the production of pro-inflammatory cytokines (IL-1β, IL-6, IL-18 and TNFα) [10], generation of ROS, endothelial expression of adhesion molecules (vascular cell adhesion molecule 1 (VCAM-1) and intercellular cell adhesion molecule 1 (ICAM-1), which promote monocyte entry into the subendothelium), increased production of endothelin-1 and reduced generation of nitric oxide (that enhance vasoconstriction) [19] and increased macrophage expression of scavenger receptors (SR: CD36 and SR class A1, which promote macrophage phagocytosis) [9]. 

AGE formation on vascular cells also determines the cross-linking of collagen molecules with an increased perivascular fibrosis, which leads to a reduction in arterial compliance and also to coronary microvascular stenosis and microaneurysms [42]. LDL glycation increases the atherogenic potential of LDL: small dense LDL particles (LDL molecules desialylated) glycation favors interactions with subendothelial proteoglycans, increasing the LDL retention time in the subendothelial space and LDL phagocytosis by macrophages to form foam cells [43]. 

Small, dense LDL are also more atherogenic because of their lower binding affinity to LDL receptors and lower resistance to oxidative stress [19]. High intracellular glucose levels activate metabolic pathways that result in reactive oxygen species (ROS) overproduction, the activation of PKC, the hexosamine pathway and the polyol pathway. The increased inflammatory response leads to endothelial dysfunction with augmented vascular permeability and impaired vasodilation as well as an associated increased risk of leukocyte/platelet adhesion, thrombosis and inflammation [9].

Hyperglycemia determines directly (through an increase in diacylglycerol) or indirectly (through the oxidative stress and the increase of ROS) increased protein-kinase C (PKC) signaling, which can upregulate NF-κB and downregulate eNOS [44], stimulate the production of cytokines, the extracellular matrix, the fibrinolytic inhibitor plasminogen activator inhibitor (PAI-1), the vasoconstrictor endothelin-1 and VEGF. These changes lead to endothelial dysfunction due to the increase of vascular permeability, basement membrane thickening, vascular occlusion and angiogenesis [5].

High intracellular glucose also results in increased hexosamine pathway flux, which leads to increased N-acetylglucosamine and upregulation of PAI-1 [45]. Finally, when intracellular glucose levels are high, there is increased polyol pathway flux. A part of the glucose is reduced to sorbitol by aldose reductase, which competes for the cofactor NADPH with glutathione reductase. This deprives the cell of reduced glutathione, an important antioxidant, with the consequent ineffective reduction of toxic aldehydes, which accumulate in the cell [45].

IR is another important factor in the pathogenesis of T2DM complications and coronary vessel damage. The metabolic consequences of IR are hyperglycemia, impaired insulin secretion, oxidative stress, hypertension, dyslipidemia and increased visceral fat deposition. The pancreas attempts to overcome insulin resistance by increasing insulin secretion. Compensatory hyperinsulinemia determines exaggerated responses in the tissues that remain sensitive to insulin, with activation of the sympathetic nervous system and the increased reabsorption of renal sodium, leading to hypertension [46]. 

Insulin resistance in fat cells leads to increased lipolysis, fatty acid release, dyslipidemia and vascular abnormalities. Visceral fat is more resistant than subcutaneous fat to the action of insulin, and the release of fatty acids from visceral fat can directly (through PKC activation) block insulin-signaling pathways, thereby, increasing insulin resistance. Obesity, especially visceral obesity, is negatively correlated with insulin sensitivity [47].

### 3.2. Epigenetic Modifications

**Figure 2:**
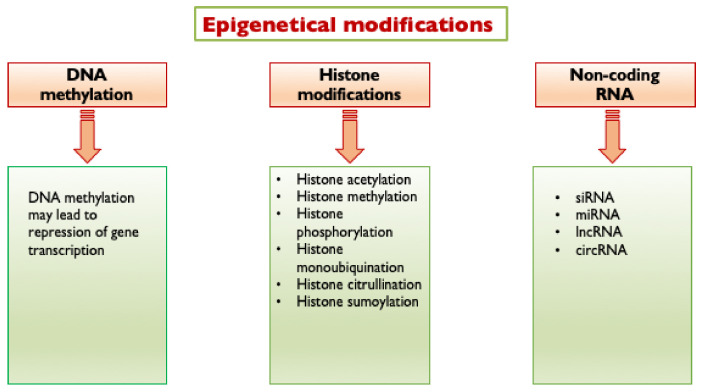
Epigenetic modifications triggered by hyperglycemia. Hyperglycemia triggers the following epigenetic modifications: DNA methylation, histone modifications and non-coding RNA mediated control. siRNA, short interfering RNA; miRNA, micro RNA; lncRNA, long non-coding RNA; and circRNA, circular RNA.

Epigenetic modifications are changes in gene function that are mitotically and/or meiotically heritable and that do not entail a change in DNA sequence [48]. They include DNA methylation, histone modifications and non-coding RNA (ncRNA)-mediated control (Figure 2). They are the result of interactions between environmental stimuli and the regulation mechanisms of DNA expression, representing a molecular link between environmental factors and complex diseases, including atherosclerosis and diabetes.

Epigenetic changes at DNA-histone complexes significantly alter gene transcription by modulating the chromatin accessibility. DNA methylation involves the addition of a methyl group to cytosines within cytosine/guanine pairs (CpG). Several types of histone modifications are known, including histone methylation, acetylation, phosphorylation, ubiquitination, SUMOylation (which is the attachment of a SUMO (Small Ubiquitin-like Modifier) peptide) and citrullination are the best studied and important in regulating chromatin structure and transcriptional activity. 

DNA methylation within a gene promoter typically represses gene transcription. Histone acetylation generally determines increased transcription, while histone methylation has either a transcriptionally permissive or repressive character, depending on the location of the targeted amino acid residues and on the number of methyl groups added [49]. In detail, the methylation of H3K9 and H3K27 is transcriptionally repressive, while the methylation of H3K4 and H3K36 is transcriptionally permissive [50].

Histone phosphorylation and histone mono-ubiquitination can regulate transcriptional activity working in conjunction (“cross talk”) with other histone modifications, such as histone acetylation [49]. SUMO peptides can use the same lysine residues as ubiquitin; therefore, the SUMOylation of substrate proteins may generate proteins resistant to degradation by the ubiquitin proteasome system [51]. 

Histone citrullination is involved in the regulation of chromatin structure and gene transcriptional activity and engages in cross-talk with histones marked with other modifications [52]. Epigenetic modifications are performed by several specific enzymes, which can be classified as “writers”, and these modify discrete residues on histone tails adding marks that designate certain regions for transcriptional regulation (such as DNA methyltransferases and histone acetyltransferases) and “erasers”, which remove these marks (such as histone deacetylases and histone demethylases) [50].

DNA methylation is catalyzed by DNA methyltransferases (DNMT) [53], whereas ten-eleven translocation (TET) enzymes are responsible for DNA demethylation [50]. Histone acetylation is regulated by histone acetyltransferases (HATs) and histone deacetylases (HDACs, further subdivided in four classes; those in class III are called sirtuins or SIRTs), while histone methylation is mediated by histone methyltransferases (HMTs) and histone demethylases (HDMs). Histone phosphorylation is controlled by kinases and phosphatases. Histone ubiquitination is conducted by histone ubiquitin ligases and histone deubiquitinating enzymes (DUBs) [49], while SUMOylation is by SUMO ligases and peptidases. Histone citrullination is mediated by peptidyl arginine deiminase (PAD) enzymes.

Epigenetic modifications are reversible, to ensure that specific genes can be expressed or silenced by specific stimulators. Shear stress, hyperglycemia, hyperlipidemia and aging are the most important factors that determinate epigenetic modifications for endothelial cells, vascular smooth muscle cell and circulating monocytes. Moreover, the epigenetic status is influenced by ncRNA molecules that regulate post-transcriptional modifications and control the mechanisms of translational inhibition [53].

### 3.3. Hyperglycemia-Induced Epigenetic Modifications and the Impact on Atherosclerosis

A growing amount of evidence suggest that, in addition to biochemical processes, hyperglycemia can also induce epigenetic modifications that lead to increased oxidative stress, PKC signaling, AGE formation, NFkB-dependent monocyte-chemotactic protein 1 (MCP-1) and VCAM1 expression, with consequent endothelial dysfunction and atherosclerosis [9].

Hyperglycemia determines DNA demethylation in endothelial cells through an upregulation of TETs. Endothelial cells exposed to transient hyperglycemia (7 days) are characterized by the persistence of NFκB-p65 gene (RELA) activation, induced by DNA demethylation of the NFκB promoter at CpGs islands through TET2 upregulation [54]. Hyperglycemia activates the NFκB-p65 gene in endothelial cells also by mono-methylation of lysine 4 on histone 3 (H3K4m1) through histone methyltransferase Set7 and demethylation of H3K9 on the p65 promoter by lysine-specific demethylase 1 [55]. 

**Figure 3:**
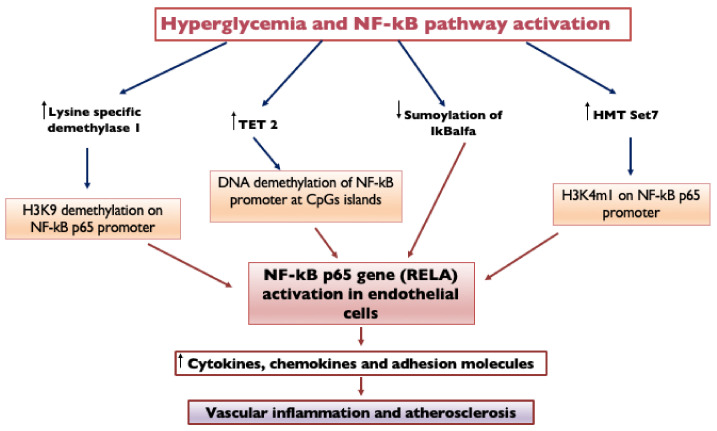
Hyperglycemia and NF-kB pathway activation. Endothelial cells exposed to transient hyperglycemia are characterized by the persistence of NFκB-p65 gene (RELA) activation, induced by DNA demethylation of the NF-κB promoter at CpGs islands through TET2 upregulation. Hyperglycemia activates the NF-κB-p65 gene in endothelial cells also by mono-methylation of lysine 4 on histone 3 (H3K4m1) through histone methyltransferase Set7 and demethylation of H3K9 on the p65 promoter by lysine-specific demethylase 1. Hyperglycemia also influences the SUMOylation of I𝜅B𝛼, which is the main inhibitor of NF-κB dimers: when IκBα is SUMOylated, it is resistant to ubiquitin-induced degradation, and it inhibits NF-κB; in high glucose conditions, SUMOylation of I𝜅B𝛼 is decreased, with consequent activation of the NF-κB pathway. NF-κB upregulation leads to increasing its gene targets, causing vascular inflammation and atherosclerosis. NF-kB, Nuclear Factor κB; TET2, Ten-eleven translocation 2; IκBα, nuclear factor of kappa light polypeptide gene enhancer in B-cells inhibitor alpha; HMT, Histone lysine methyltransferase; H3K9 demethylation, Demethylation of Histone H3 at Lysine 9; and H3K4m1, Monomethylation on lysine 4 of histone H3.

Hyperglycemia also influences the SUMOylation of I𝜅B𝛼, which is the main inhibitor of NF-κB dimers: when IκBα is SUMOylated, it is resistant to ubiquitin-induced degradation, and it inhibits NF-κB. In high glucose conditions, the SUMOylation of I𝜅B𝛼 is decreased, with consequent activation of the NF-κB pathway [56]. The NFκB upregulation leads to increasing its gene targets, including adhesion molecules, cytokines and chemokines (such as VCAM1, IL-6, TNFα and MCP-1), causing vascular inflammation and atherosclerosis (Figure 3). Ubiquitination and SUMOylation can also activate TGF-β and MAPK and inhibit Nrf2-oxidative stress [51]. Ubiquitin-proteasome system overactivity, by contributing to the increased inflammation process, may enhance the risk of complication during myocardial ischemia in diabetic patients [57].

The activation process of Set7 takes also place in circulating monocytes, determining the methylation of H3K4m1 on the NFκB-p65 promoter, with activation of the transcription of genes responsible for the inflammatory process and for the increased adhesion capacity of monocytes to the endothelium, leading to vascular dysfunction [58]. Hyperglycemia is the most potent driver of Set7 upregulation, and the expression of Set7 in circulating monocytes may represent a potential marker of vascular damage. Targeting this chromatin-modifying enzyme may represent a novel therapeutic approach [58].

High glucose also induces DNA demethylation of the superoxide dismutase 2 (SOD2) promoter through TET2 upregulation, leading to reduced SOD2 expression, increased oxidative stress and vascular dysfunction [54].

Hyperacetylation of histone H3 at lysine residues 9 and 14 (H3K9/K14) coexisting with DNA hypomethylation of CpG islands was observed in response to hyperglycemia in human vascular cells on multiple gene promoters, including heme oxygenase 1 (HMOX1), IL-8, matrix metalloproteinase 1 (MMP1) and 10 (MMP10) and cysteine/glutamate transporter (SCL7A11) [59]. Chromatin immunoprecipitation assays in monocytes demonstrated that diabetic patients present an increased acetylation of histone H3 at lysine residues 9 and 14 and of histone H4 at lysine residues 5, 8 and 12 of cyclooxygenase 2 (COX2), TNFα and MCP-1 gene promoters, causing enhanced inflammatory genes transcription and monocyte activation [60]. These genomic regions with high amounts of H3K9/14 histone tail hyperacetylation represent 0.3% of the genome in cells cultured in hyperglycemic conditions [59].

Hyperglycemia also induces TET-2 mediated DNA demethylation changes that are involved in the differentiation of the vessel smooth muscle cells in a phenotype characterized by loss of contractility and increased proliferation and secretion of extracellular matrix proteins that promote atherosclerosis. TET2 knockdown determines the reduced expression of vascular smooth muscle cells (VSMCs) contractile genes (such as MYOCD, SRF and MYH11 genes), increased the expression of synthetic phenotype markers, such as KLF4, KLF5 and OPN genes and the increased proliferation of human coronary artery VSMCs [61]. A U.K. Prospective Diabetes Study showed that end-organ effects continued to operate >5 years after the patients had returned to their usual level of glycemic control because of the “hyperglycemic memory” or “legacy effect” [62]. 

Transient hyperglycemia and the relative induced epigenetic modifications are able to determine a persistent activation of genes harmful to the cardiovascular system, even when glycemic control is reached and maintained. Human aortic endothelial cells exposed for 7 days to high glucose presented lower DNA methylation levels for the SIRT6 promoter with increased expression of SIRT6 and TET2; these epigenetic changes persisted after 48 h of glucose normalization [63]. The increased SIRT6 expression following high-glucose exposure might be a compensatory response to hyperglycemia-induced damage, since SIRT6 is involved in the DNA-damage repair system and protects against endothelial dysfunction and vascular inflammation [64].

Epigenetic changes in the promoter of NF-κB subunit p65 (increased H3K4m1 by the histone methyltransferase Set7) in aortic endothelial cells induced by transient hyperglycemia (16 h) persist for at least 6 days of subsequent normal glycemia, as do NF-κB-induced increases in MCP-1 and VCAM-1 expression [65]. H3K4 methylation of NF-κB p65 subunit also provides a mechanism for epigenetic memory in macrophages [66].

Short-term (72 h) high glucose stimulation induces persistent downregulation of deacetylase SIRT1 as well as the upregulation of acetyltransferase p300, leading to sustained hyperacetylation (at K382) and activation of p53 and subsequent p53/p21-mediated senescent “memory” [67].

Hyperglycemia is not the only trigger of epigenetic modifications. AGEs directly reduce SIRT1 expression, leading to an increased acetylation process and to the activation of p53, which is responsible for apoptosis and endothelial dysfunction [68]. Furthermore, the products of oxidative processes induced by hyperglycemia determine p66Shc promoter DNA demethylation and GCN5-mediated histone 3 hyperacetylation. These epigenetic modifications lead to p66Shc overexpression, which supports ROS production and antioxidant manganese superoxide dismutase downregulation, resulting in unchallenged ROS accumulation in the vascular endothelium and endothelial dysfunction [69]. 

ROS production activates PKCβII, which in turn maintains elevated p66Shc levels, promoting sustained ROS-dependent epigenetic changes. Hyperglycemia-mediated ROS overproduction is indeed considered the major driver of glycemic memory in the vasculature [50]. Some authors hypothesized a two-stage mechanism for hyperglycemic memory: an initial induction phase due to hyperglycemia-induced increased ROS production and a perpetuation phase, since ROS induces mutations in the mitochondrial DNA with the encoding of defective electron transport chain subunits that cause increased ROS production by the electron transport chain also at physiologic concentrations of glucose and glucose-derived reducing equivalents [5]. 

SIRT1 deacetylates H3 histones at the human endothelial p66Shc promoter to suppress the transcription of ROS and promote transcription of SOD; therefore, SIRT1 prevents hyperglycemia-induced endothelial dysfunction and avoids hyperglycemic memory [70]. Adipose tissue from insulin-resistant subjects presents DNA hypermethylation of different genes, such as FTO (associated with increased risk of obesity and cardiovascular diseases) [71] and IGF2 (associated with a higher triglyceride/HDL ratio and increased metabolic risk in children) genes [72].

Low-grade chronic inflammation and insulin resistance induce histone deacetylase 3 (HDAC3) activity and expression in peripheral blood mononuclear cells [73]. A HDAC3 increase is associated with a decreased transcriptional level of DCB1, which negatively regulates HDAC3. HDAC3 activity correlates with increased TNFα, IL-6, MCP-1 and reduced SIRT1, which causes macrophage recruitment and infiltration into adipose tissue [73]. In obesity-induced insulin resistance/T2DM, the elevated levels of saturated fatty acids (SFAs) enhance DNA methyltransferase 3b activity, leading to the methylation of the PPRγ promoter and triggering the macrophage phenotype switch from anti-inflammatory (M2) to pro-inflammatory (M1) [74].

### 3.4. Micro-RNA, Long Non-Coding RNA and Circular RNA Impacts on Atherosclerosis and DM

Regulatory non-coding RNAs include small interfering RNAs (siRNAs), micro-RNAs (miRNAs), long-non-coding RNAs (lncRNAs) and circular RNAs (circRNAs). siRNAs and miRNAs are short (20–22 nucleotides length) fragments that regulate gene expression at a post-transcriptional level. siRNAs and miRNAs both target mRNA producing a gene silencing effect; however, siRNAs inhibit one specific target mRNA, while miRNAs regulate the expression of multiple mRNAs at the same time [75]. miRNAs mediate several cellular processes by inhibiting their targets through either translational repression or mRNA degradation. 

LncRNAs are >200 nucleotides in length and are involved in the regulation of transcription, splicing and in the regulation of miRNAs [76]. CircRNAs are covalently closed circular molecules, formed by joining of the 3′ to the 5′ splice site, which can arise from any region of the genome, including exonic circRNAs (ecircRNAs), intronic circRNAs (ciRNAs) and exon–intron circRNAs (EIciRNAs). ecircRNAs mostly interact with miRNAs in the cytoplasm, while ciRNAs and EIciRNAs are involved in the regulation of gene transcription at the nuclear level [77].

Dysregulated expression of different miRNAs and lncRNAs is involved in various steps of atherosclerosis, including altered lipid metabolism, endothelial dysfunction, vascular smooth cell and macrophage phenotypic switching, platelet reactivity and cardiomyocyte differentiation and apoptosis [9]. More than 2500 miRNAs were identified in the human genome, and several of them were found to be involved in diabetes mellitus pathogenesis, insulin sensitivity, cardiomyocytes hypertrophy, fibrosis and diastolic dysfunction [19]. Different miRNAs were found to play important roles for both T2DM and CVD. 

Hyperglycemia upregulates miRNA-185 with reduction of the GPx-1 gene, which encodes the glutathione peroxidase-1 enzyme, which is important in preventing oxidative stress and endothelial dysfunction [78]. The upregulation of miRNA-34a [79] and miRNA-204 [80], which impair SIRT1, and the reduction of miRNA-126, which plays a protective role in atherosclerosis, are involved in endothelial dysfunction in patients with DM and CAD [81]. SIRT-1-impaired expression contributes to endothelial cell senescence, while miR-126 reduction determines the loss of inhibition of TNFα, the ROS level and NADPH oxidase via HMGB1 [82] and the loss of stimulation on VEGF and fibroblast growth factor activities, with consequent pro-inflammatory and anti-angiogenetic effects [27].

Hyperglycemia also downregulates the expression of miRNA-130a and miRNA-134, altering endothelial cells apoptosis and motility. miRNA-130a expression inhibition leads to increased Runx3 and decreased VEGF, with consequent increased apoptosis and decreased proliferation, migration, differentiation, colony formation and angiogenic potential [83]. In the presence of hyperglycemia, miRNA-320 is upregulated and suppresses the VEGF-A, FGFs, IGF-1 and IGF-1 receptors, with reduced proliferation and migration and impaired angiogenesis [84]. 

Endothelial cells incubated in high glucose also present an increased expression of other miRNAs involved in impaired angiogenesis, such as miRNA-221, 222 and 503. miRNA-221 alters the expression of c-kit (CD117), the receptor for stem cell factors, impairing endothelial progenitor cell migration and homing [85]; in addition, both miRNA-221 and 222 directly inhibit cyclin-dependent kinase inhibitor proteins P27KIP1 and P57KIP2, inhibiting the cell cycle [86]. miRNA-503 determines the reduced expression of the cell cycle regulators cdc25A and cyclin E1 (CCNE1), determining an impaired cell cycle [87].

High glucose levels also determine miRNA-145 reduction and miRNA-24 and miRNA-504 increases, with the consequent VSMC acquisition of proliferative and migratory properties. In response to high glucose stimulation, VSMCs also present increased expression of miRNA-125b, which decreases H3K9 methylation at inflammatory gene promoters by targeting methyltransferase Suv39h1 mRNA, leading to increased IL-6 and MCP-1 gene expression [88]. This demonstrated the role of miRNA-125b in accelerating atherosclerosis. 

There are also miRNAs involved in lipid metabolism dysregulation (increased miRNA-29 and miRNA-122 and reduced miRNA-26a levels determine abnormal cholesterol homeostasis and fatty acids synthesis) and platelet function alteration (downregulation of miRNA-223, 126 and 146a, associated with increased platelet reactivity and the upregulation of miRNA-103b) [89].

miRNAs serum levels have been shown to be stable, reproducible and consistent amongst healthy individuals; therefore, they have potential value as diagnostic and prognostic biomarkers of disease. For example, decreased miRNA-126 plasma levels were associated with a low ankle brachial pressure index and subsequent new-onset peripheral vascular disease [90].

Different lncRNAs have been implicated in various steps of atherosclerosis and insulin-resistance processes. lncRNAs influence DM-associated cardiovascular disease by regulating endothelial cell growth, migration and apoptosis. In hyperglycemic conditions, endothelial dysfunction is mediated by the upregulation of lcnRNA MALAT1 (which targets the proinflammatory ligand serum amyloid antigen 3, inducing the expression of IL-6 and TNFα and ROS production), lncRNA CDKN2B-AS1 (ANRIL, which affects CVD progression by inducing VSMCs proliferation and cell adhesion and reducing VSMCs apoptosis) and MIAT (retinal non-coding RNA, implicated in endothelial cells migration by targeting miRNA-150-5p, causing increased TNFα and VEGF expression) and downregulation of MEG3 (involved in endothelial cell proliferation, migration and tube formation via PI3K/Akt) [91].

Other lncRNAs play important roles in other cells involved in the process of atherosclerosis induced by hyperglycemia. In VSMCs, lncRNA H19 upregulation increases proliferation through its target IGF, while lncRNA-p21 downregulation stimulates proliferation and reduces apoptosis (via p53) [91]. In macrophages, lncRNA E330013P06 upregulation promotes foam cell formation through the expression of inflammatory genes, such as Nos2, IL6 and ptgs2 [92], while lncRNA-Lethe reduction determines an increased inflammation cascade removing the inhibition on the translocation of NF-κB transcription factors to the nucleus, with a consequent increase in NOX2 gene expression and ROS production [93]. MALAT1, lncRNA-GAS5 and lncRNA-p21 (via p53) are involved in macrophages apoptosis [91]. Finally, lncRNA H19 downregulation is associated with cardiomyocytes dysfunction (lncRNA H19 via miRNA-675 targets VDAC1, a mitochondrial porin involved in ATP transport, leading to cardiomyocyte apoptosis) [94].

CircRNAs are involved in physiopathologic processes as they can serve as miRNA sponges, regulate transcription or splicing and can also interact with RNA binding proteins (RBPs) [95]. CircRNAs play an important role in the initiation and development of atherosclerosis by regulating the activation of endothelial cells, VSMCs and macrophages by targeting miRNA [77].

Ox-LDL-treated vascular endothelial cells present upregulation of Circ-0003575, which is involved in endothelial cell proliferation and angiogenesis [96]. High glucose treated endothelial cells present downregulation of circHIPK3 that acts through the axis miRNA-30a-3p-VEGFC/WNT2/FZD4, with a consequent lack of inhibition of oxidative damage that causes cell apoptosis and of circDNMT3B that, via miRNA-20b-5p/BMP and the BAMBI axis, leads to increased angiogenesis. In addition, endothelial cells treated with high-glucose also present upregulation of circ-0054633 (which acts via miRNA-218/ROBO1 and HO-1) and of circ-0068087 (which acts through miRNA-197/TLR4/NF-κB/NLRP3), leading to increased angiogenesis and inflammation [77].

CircRNAs are also involved in VSMC proliferation and migration in conditions of elevated ox-LDL levels (circCHFR) and hyperglycemia (circWDR77- miRNA124-FGF2 pathway) [97], neointimal hyperplasia on endothelial and vascular smooth muscle cells (circDiaph3), atherosclerotic plaque calcification by increased release of hydroxyapatite (circSp140 and circVxs1), induction of the endothelial–mesenchymal transition process (circHECW2 and circDLGAP4) and macrophage differentiation and polarization in M1 pro-inflammatory type (circRNA-003780, circRNA-010056 and circRNA-010231) [77]. 

circRNA11783 -2 is closely related with both atherosclerosis and type 2 diabetes, and it acts as an miRNA sponge on miRNA-608 and miRNA-3907 [98]. CircRNAs are more stable than linear RNAs in plasma and body fluids; therefore, they could have a role not only as target therapy but also as early diagnostic and prognostic markers of atherosclerosis [77].

## 4. Role of Epidrugs in DM-Induced CAD

**Figure 4:**
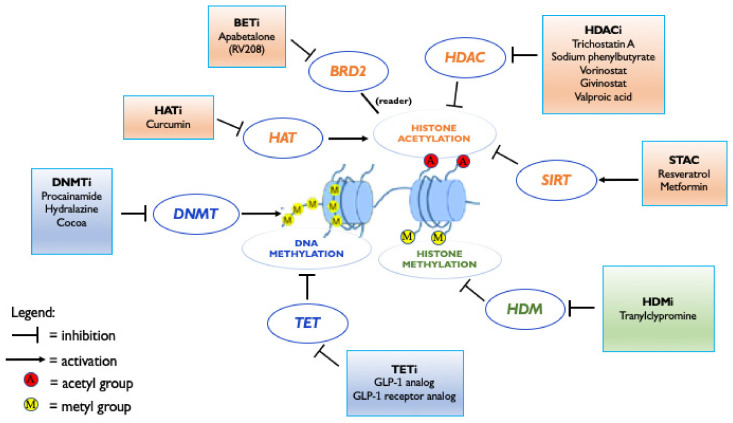
Main classes and mechanisms of action of epidrugs. The main classes of epigenetic modifiers include DNA methyltransferase inhibitors (DNMTi), histone demethylating inhibitors (HDMi), histone deacetylase activators (and in particular class III, sirtuin-activating compounds, or STACs), histone deacetylase inhibitors (HDACi), histone acetyltransferase inhibitors (HATi) and bromodomain and extra-terminal domain inhibitors (BETi). DNMT, DNA methyltransferase; TET, ten-eleven translocation enzymes; HDM, histone demethylase; HAT, histone acetyltransferase; HDAC, histone deacetylase; SIRT, sirtuins; BRD2, bromodomain 2; and GLP 1, glucagon-like peptide 1.

In patients with cardiometabolic diseases, such as DM, it is possible to restore the altered epigenetic landscape by the administration of compounds able to edit/erase specific biological signals. In the recent years, several drugs targeting epigenetic modifications have been developed and named “epidrugs”. The main classes of epigenetic modifiers include DNA methyltransferase inhibitors (DNMTi), histone demethylating inhibitors (HDMi), histone deacetylase activators (HDACa), sirtuin-activating compounds (STACs), histone deacetylase inhibitors (HDACi), histone acetyltransferase inhibitors (HATi) and bromodomain and extra-terminal domain inhibitors (BETi) (Figure 4).

### 4.1. Antidiabetic Drugs with Epigenetic Action

Interindividual variation to antidiabetic medication response can be only partially explained by genomic diversity, and epigenetics can help explain some of the variability in the dose requirements and adverse effects of oral antidiabetic drugs. Some beneficial effects of antidiabetic drugs to the endothelium can be attributed to the inhibition of diabetes-induced epigenetic changes [53]. Metformin, a biguanide compound, is a well-established antidiabetic drug in T2DM with proven effects of reducing rates of cardiovascular disease and death in diabetic patients. Metformin acts on hyperglycemia by decreasing hepatic gluconeogenesis and increasing insulin sensitivity. 

The protective effect of metformin on vascular cells occurs in part via glucose lowering and in part via other pleiotropic effects, including DNA methylation. Metformin treatment leads to a combination of hyper-, hypo- and non-differentially methylated CpG sites, and this is due to a combination of direct and indirect effects [99]. 

Patients on low-dose metformin therapy were found to present several differentially methylated genes compared to healthy individuals, including CAMKK1 (a regulator of AMPK), BACE2 and ADAM8 (which regulates monocyte mobility and inflammation). Since this study compared regions with significant differences in methylation levels between healthy subjects and patients on metformin therapy, the observed DNA methylation changes induced by metformin were independent of diabetic status [100]. Metformin delays endothelial senescence enhancing mitochondrial biogenesis by AMPK-SIRT1-DOT1L-mediated H3K79 trimethylation of SIRT3 promoter, causing SIRT3 increased expression and reduced oxidative stress. 

Chronic low-dose metformin treatment increases the mitochondrial oxygen consumption rate, significantly attenuates vascular aging and inhibits age-associated atherosclerotic plaque formation [101]. Metformin-mediated enhancement of SIRT1 signaling (SIRT1/p300/p53/p21) also provides protection against metabolic memory of cellular senescence [67]. Metformin also acts by upregulating miRNA let-7 through AMPK activation, leading to lncRNA H19 degradation, S-adenosylhomocysteine hydrolase activation, DNMT3B activation and DNA methylation of a subset of genes [102].

Modulation of epigenetic factors might be an additional mechanism by which glucagon-like peptide 1 (GLP-1) agonists and GLP-1 receptor agonists could prevent diabetic complications. GLP-1 agonists and exenatide (GLP-1 receptor agonist) prevent high glucose-induced DNA demethylation changes on NF-kB and SOD2 promoter regions by reducing the binding of TET2 to these regions. As a result, these drugs determine a reduction of NF-κB activation and NF-κB target genes (IL-6, TNFα, VCAM1) expression and an increase of SOD2 expression, with a favorable effect in terms of vascular inflammation [54].

Recently, a new class of anti-diabetic drugs, sodium-glucose cotransporter-2 inhibitors (SGLT2i), have been proposed by the most recent ESC guidelines as first-line therapy in patients with HF [103]. The efficacy of these drugs in the improvement of cardiac function is also through their epigenetic effects. Solini et al. suggested novel mechanisms of cardioprotection associated with dapaglifozin treatment. Dapaglifozin upregulates miRNA-30e-5p, which inhibits myocardiocyte autophagy and heart failure onset and reduces miRNA-199a-3p expression, which causes a reduction in cardiac PPARδ levels, thus, restoring mitochondrial fatty acid oxidation and leading to an improvement of cardiac function in patients with HF. Dapaglifozin also exerts nephroprotection by preserving renal vasodilating capacity through a reduction in miRNA-27b expression [104].

### 4.2. Phytochemicals with Epigenetic Mediated Effects on Diabetes-Induced CAD

Currently, there are approximately 200 pure bioactive compounds that can be found in medicinal plants with potent hypoglycemic effects [105] through DNA-methylation and histone modifications. Several of these compounds have already been approved by the FDA for the treatment of cancer and neurological diseases [106]. The applicability of major phytochemical groups, such as polyphenols, terpenoids, organosulfur and alkaloids as epidrugs in patients with diabetes was experimentally shown [107]. An extensive description of phytochemicals with epigenetic effects on diabetes goes beyond the scope of this review, but here we provide some examples.

Resveratrol is a natural compound (phytoalexin stilbene) that is highly concentrated in grapes, red wine, berries and peanuts. This phenolic molecule is a potent activator of NAD-dependent HDAC sirtuin 1 (SIRT-1) [108] and it is able to inhibit AGE-induced apoptosis in human vascular endothelial cells by decreasing the expression of apoptosis-associated signaling molecules, such as cytochrome c [68]. Resveratrol inhibits high glucose-induced metabolic memory of endothelial senescence (through the SIRT1/p300/p53/p21 pathway) [67]. Through SIRT1, resveratrol also reduces endothelial oxidative stress by the activation of Nrf2, decreased NF-κB expression, suppression of pro-oxidative genes (such as NADPH oxidase subunit Nox4) and induction of anti-oxidative enzymes (such as superoxide dismutase 1 and glutathione peroxidase 1) [109]. 

The resveratrol antioxidant effect improves insulin sensitivity (through the Akt pathway) and glucose control in T2DM patients [110]. Therefore, resveratrol may be an alternative therapy to treat T2DM-associated vascular complications [68,110]. Different clinical trials investigated the role of resveratrol in T2DM [111], diabetic microvascular complications [112], atherosclerosis, coronary artery disease and peripheral artery disease [113], and several studies are currently ongoing. RESPECT-ISR (NCT05093244) is a prospective, randomized multicenter study that will investigate a resveratrol excipient paclitaxel-coated balloon for the treatment of in-stent coronary artery restenosis.

Curcumin is the active ingredient of curcuma longa, a common spice used in curry dishes [114]. Curcumin supplementation was shown to exert beneficial effects through specific inhibition of histone acetyltransferase p300 with the consequent inhibition of p53, leading to the induction of angiogenesis and inhibition of NF-κB activation, with significant reduction in pro-inflammatory cytokines, TNFα, TGFβ1, ROS, MCP-1, increase in NO synthase and improvement of microangiopathy, endothelial senescence and hyperglycemic memory [114,115]. Curcumin also attenuates TNFα-mediated lipolysis and is associated with decreased levels of circulating free-fatty acids and improved insulin sensitivity [116]. Supplementation of curcumin reduced serum cholesterol in atherosclerotic patients [117], and a randomized clinical trial in T2DM patients showed that a 6-month curcumin intervention significantly reduced the atherogenic risk [118].

Apabetalone (RVX 208) is a selective bromodomain and extra-terminal (BET) protein inhibitor targeting bromodomain2 (BD2); it is involved in gene transcription regulation by interacting with acetylated histone residues, and it is therefore considered an epigenetic “reader”. BD2-selective BET inhibition by apabetalone suppresses TNFα-induced expression of vascular inflammation genes. Treatment with apabetalone through its epigenetic effects reduces mediators that drive endothelial activation, monocyte recruitment and plaque destabilization [119]. 

In phase II trials, Apabetalone reduced the relative risk of major adverse cardiac events (MACE) in diabetic coronary artery disease patients by 57% compared to placebo [120]. Recently published data of a phase III clinical trial (BETonMACE) on high-risk T2DM patients with coronary artery disease failed to clearly demonstrate a reduction in MACE but showed promising effects of Apabetalone on specific secondary endpoints, such as cardiovascular death and non-fatal MI and HF [121].

### 4.3. Non-Coding RNA-Based Therapeutics

An important family of developing epidrugs includes ncRNA-based therapeutics that are currently in preclinical development, some of which are entering assessment in clinical trials [122]. Although there is no direct evidence to utilize ncRNAs in clinical settings for the CVD complications in DM, many experimental studies suggest that ncRNAs might be used in the future as therapeutic agents for the treatment of vascular complications developing from DM [91]

Endogenous miRNAs and lncRNAs can be inhibited through the administration of antisense oligonucleotides that are complementary to their sequences. Smaller ncRNAs, such as siRNAs and miRNAs, can also be delivered through chemical vectors (such as lipid mixtures, lipid nanoparticles and dendrimers) and biological vehicles, including exosomes and minicells. RNA therapeutics can also be delivered through viral vectors [9]. miRNA therapeutics include miRNA mimics, miRNA sponges, anti-miRNA oligonucleotides and anti-sense oligonucleotides. miRNA mimics are able to mimic the function of an endogenous miRNA, while miRNA sponges bind to intracellular miRNAs, preventing its binding to targets. Anti-miRNA oligonucleotides (antagomiRs) form a stable, high-affinity bond with the miRNA, reducing the availability of the latter to bind its target [123]. 

Anti-sense oligonucleotides (ASOs) hybridize with the complementary mRNA in a sequence-specific manner via Watson–Crick base pairing; the ASO-mRNA heteroduplex can lead to mRNA degradation through RNase-H activity, translational arrest by steric hindrance of ribosomal activity, or downregulation of target protein expression by inhibiting splicing or destabilizing pre-mRNA in the nucleus [124].

A promising miRNA therapeutic approach in the cardiometabolic disorders field is testing the use of an anti-miRNA–33, since miRNA-33 suppresses the expression of the cholesterol transporter ABC transporter A1 (ABCA1) and lowers HDL levels. Rotllan et al. observed a reduction of aortic atherosclerotic lesion in mice after 8 weeks of anti–miRNA-33 treatment [125]. The targeted deletion of miRNA33 in Apoe–/– mice determines the reduced atherosclerotic plaque size, increased markers of plaque stability, decreased inflammatory gene expression and increased HDL plasma levels [126].

AntagomiR targeting miRNA-195 has entered the preclinical development stage for treating diabetic cardiomyopathy [127]. miRNA-195 silencing increases the levels of the antiapoptotic protein BCL-2 (preventing cardiomyocytes apoptosis) and of SIRT1 (regulating cardiomyocyte survival). miRNA-195 silencing attenuates diabetic cardiomyopathy also by reducing oxidative stress and mRNA levels of hypertrophic genes and by increasing coronary blood flow and myocardial capillary density [127].

Anti-miRNA21-coated drug-eluting stents have been shown to be highly effective in reducing VSMC proliferation and alleviating myointimal hyperplasia and in-stent restenosis in humanized animal models of obstructive artery disease, and this did not show any off-target side effects, in contrast to the systemic use of anti-miRNA 21. In addition, in contrast to currently employed DES, it did not delay vessel re-endothelization and did not inhibit endothelial cell proliferation in vitro, likely because of the low concentration of mi-RNA 21 in the endothelium compared to its expression in VSMCs in response to injury and stent deployment [128].

Mipomersen is an anti-sense oligonucleotide that inhibits the synthesis of ApoB mRNA in the liver and currently has approval for the treatment of familial hypercholesterolemia (FH). Phase 3 clinical trials documented that long term mipomersen treatment leads to a reduction in cardiovascular events in FH patients [129].

Another possible therapeutic strategy is represented by locked nucleic acids (LNA)-GapmeRs that bind lncRNAs and activate RNase H with the consequent cleavage and downregulation of specific lncRNAs that are over-expressed in pathological conditions. Kuespert S. et al. experimented in vitro with antisense oligonucleotides in an LNA-GapmeR-based technology that blocks TGFβ signaling by inhibiting TGFβ receptor II mRNA [130]. This suggests a promising future role of LNA-GapmeRs as regulators of lncRNA expression implicated in other signaling pathways in the contest of different pathologies, including diabetes and atherosclerosis.

Another kind of technology could be the use of nanoparticles to deliver into target cells specific lncRNAs that are downregulated in pathological conditions. Although the role of lncRNAs is promising in the therapeutic field, preclinical studies are limited: many lncRNAs are not conserved between species, and this reduces the possibility of translating the results of preclinical studies to clinical studies.

### 4.4. Challenges and Limitations of Drugs with Epigenetic Targets

The development of drugs with epigenetic targets presents different limitations, including the fact that epigenetics are tissue specific and environment- and time-dependent. In addition, it is difficult to prove a causal relationship between epigenetic changes and the disease phenotype. Therefore, epigenetic studies require much work before they can be translated into clinical practice, and this requires expensive laboratories and techniques where research can be performed [53]. Approaches that allow plaque- and cell-specific pharmacological targeting of chromatin regulators, such as a nanomedicine delivery strategies that use drug-loaded high-density lipoprotein nanoparticles, may help in developing greater precision epidrugs [131].

NcRNA therapeutic approaches face several challenges, ranging from ncRNA drug design and delivery to regulatory issues. In order to be of potential use, there should be a predominant pathomechanism addressed with no irreversible damage present, and the drug has to be efficient with very high safety [122]. NcRNAs can also be used as diagnostic tools for earlier disease detection and differentiation of disease subtypes and as prognostic markers. For example, a study from Meder et al. identified a unique 20-miRNA signature from the total peripheral blood that was able to predict AMI with high specificity and sensitivity values, when troponin was still negative [132]. More recently, the lncRNAs CoroMarker and PPARδ were reported as predictive biomarkers for CAD [133].

Research on ncRNAs as biomarkers faces several challenges that include the variability related to the choice of material, as ncRNA levels greatly vary among the different materials used, RNA-extraction methods and processing techniques. The results may be affected by the influence of other drugs (e.g., heparin has been shown to interfere with circulating ncRNAs levels [131]) and other non-cardiac diseases. Moreover, the current normalization methods for reporting ncRNA levels are not standardized. 

Therefore, it is necessary to eliminate the variability of ncRNA data due to technical and analytical factors as standardization is needed to generate solid and reproducible results. This represents a major challenge to refine the search for ncRNAs as biomarkers [122]. LncRNA research is also challenging due to the fact that most lncRNAs, in contrast to miRNAs, are not conserved among species; therefore, the translation of animal findings to patients will be challenging [122].

## 5. Conclusions

Diabetic patients present an increased risk of coronary heart disease; however, the pathogenesis of CAD in diabetes mellitus is still not completely understood. Epigenetic modifications can help understand the pathophysiological link between diabetes and accelerated atherosclerosis; epigenetics also have a role in hyperglycemia-linked biological memory and can help clarify differences in antidiabetic drug responses among individuals of the same genotype group.

Increased knowledge of epigenetic mechanisms has opened new perspectives on the diagnostic, prognostic and therapeutic applications of epigenetics in cardiovascular diseases. New epigenetic biomarkers may help to predict and detect the development and progression of diabetes complications at an early stage, thus, allowing early intervention in order to delay severe complications. Further studies on diabetes and CAD epigenetics are needed to improve the clinical use of antidiabetic drugs in DM-induced CAD, to identify novel therapeutic targets and to predict patients who will benefit from therapy, thereby, maximizing the effective therapy and minimizing the incidence of adverse effects.

## Data Availability

No new data were created or analyzed in this study. Data sharing is not applicable to this article.
